# Vascular Diameters as Predictive Factors of Recanalization Surgery Outcomes in Internal Carotid Artery Occlusion

**DOI:** 10.3389/fneur.2021.632063

**Published:** 2021-09-06

**Authors:** Chengrui Yan, Jiaru Wang, Ruohan Guo, Weitao Jin, Yang Zhao, Rong Wang

**Affiliations:** ^1^Department of Neurosurgery, Peking University International Hospital, Beijing, China; ^2^Department of Radiology, Peking Union Medical College, Peking Union Medical College Hospital, Chinese Academy of Medical Sciences, Beijing, China; ^3^Peking Union Medical College and Chinese Academy of Medical Sciences, Beijing, China; ^4^Department of Neurosurgery, Beijing Tiantan Hospital Capital Medical University, Beijing, China

**Keywords:** outcome research, recanalization, internal carotid artery occlusion, high-resolution vessel wall magnetic resonance imaging, endovascular intervention, carotid endarterectomy

## Abstract

**Background:** Revascularization surgery sometimes can achieve recanalization in patients with internal carotid artery occlusion (ICAO). High-resolution vessel wall magnetic resonance imaging (HRVWI) is a feasible technique to give detailed characteristics of the vessel wall, which may help to identify patients that carry higher success rates and more suitable for revascularization surgery.

**Objective:** To examine the association between HRVWI characteristics of ICAO and the success rate of revascularization surgery in ICAO patients.

**Methods:** We conducted a retrospective analysis of 31 ICAO recanalization patients enrolled from October 2017 to May 2019. The clinical data of patients and lesions were collected and analyzed.

**Results:** A total of 31 ICAO patients were enrolled in this study. No significant differences were found between recanalization success and recanalization failure groups with regard to occlusion length, distal end of the occluded segment, and the treatment applied. The ipsilateral-to-contralateral diameter ratios (I/C ratios) of C1 or C2 and the diameter of C7 were positively related to recanalization success. A two-factor predictive model was constructed, and the I/C ratio of C2 < 0.86 and the diameter of C7 < 1.75mm were separately assigned 1 point. The ICAO patients who scored 0, 1, or 2 points had a risk of 5.6% (1/18), 55.6% (5/9), or 100% (4/4) to fail in the recanalization.

**Conclusions:** The I/C ratios of C1 or C2 and the diameter of C7 are predictive factors of a revascularization surgery success in ICAO patients. A risk stratification model involving C2 and C7 was constructed for future clinical applications.

## Introduction

Chronic internal carotid artery occlusion (ICAO) was usually formed based on progressive atherosclerosis at the bifurcation of the carotid artery (1). Progressive stenosis of internal carotid artery (ICA) could reduce the blood flow in the ICA perfusion area, potentially leading to stroke. However, extracranial-intracranial or intracranial collateral circulation established during the progression could compensate the compromised perfusion, which accounts for the fact that some patients are asymptomatic despite severe ICA stenosis or ICAO. Both symptomatic and asymptomatic ICAO patients are at high risk for stroke. Faught et al. (2) reported that the 4-year cumulative stroke rate of asymptomatic ICAO patients or patients with transient ischemic attack was 8–11%, while in patients with apoplectic carotid artery occlusion, the risk was a higher 33%, which was still as high as 12.5% even after tPA therapy (3).

Carotid endarterectomy (CEA) could directly revascularize the narrow or occluded ICA and improve intracranial blood flow. It applies to the cases with a short occlusive length in the extracranial part of ICA, with a recanalization rate of 40.7–87.5% (4). Meanwhile, endovascular treatment could be used to recanalize long occlusive lesions with a success rate of 61.6–88% (5, 6). For ICAO patients with distal occlusion, such as the clinoid segment and above, Liu et al. reported that the recanalization rate of hybrid treatment was 71.4% (7). Therefore, the combination of CEA and endovascular treatment, or hybrid treatment, is a feasible therapy. However, endovascular recanalization of ICAO is still technically challenging due to long occlusion length and wide individual variation of the occluded vessel course. Potential complications after wiring injury, including hemorrhage, pseudoaneurysm, and carotid-cavernous fistula, might be catastrophic. Therefore, a systematic pre-procedural evaluation is important to identify patients that carry a higher recanalization success rate. According to previous studies, length of occlusion, occlusion duration, plaque location, and distal ICA reconstitution at a higher segment might affect the success rates (3). However, these are all qualitative indicators, and quantitative indicators to predict the success rate are yet to be found.

Recently, high-resolution vessel wall magnetic resonance imaging (HRVWI) emerged as a practicable technique to visualize luminal thrombi and vessel wall in occluded ICA (8). We found that the diameters of the occluded vessel could predict the successful rate of recanalization. This study sought to examine the predictive value of the diameter of each segment of occluded ICA in terms of achieving carotid revascularization.

## Methods

### Patients

We conducted a retrospective analysis of 31 ICAO recanalization patients who were treated at Peking University International Hospital, Beijing, China, from October 2017 to May 2019. Inclusion criteria were as follows: (1) patients should be over 18 years old; (2) diagnosed as symptomatic total occlusion or near-occlusion of the carotid artery by digital subtraction angiography (DSA); (3) the latest stroke occurred more than 8 weeks previously, and patients with more than two ipsilateral cerebral ischemia were given optimal medical treatment; (4) patients should have accepted a high-resolution vessel wall magnetic resonance imaging (HRVWI) examination with contrast before the procedure. All subjects were fully informed and gave written consent before they were enrolled in the study. This study was approved by the institutional review board of Peking University International Hospital.

**Figure 1:**
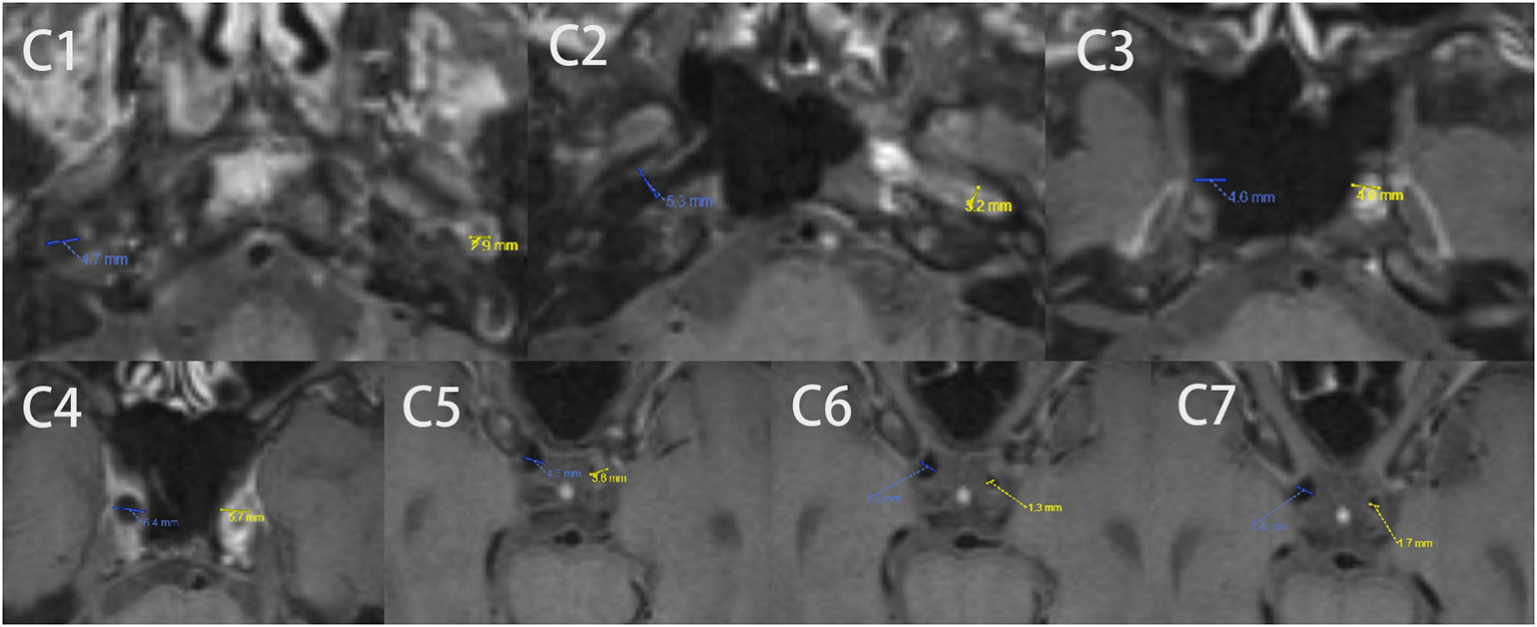
A typical example of C1-C7 diameter measurements in the axial image by HRVWI of ICAO patients. High-resolution vessel wall magnetic resonance imaging (HRVWI) examination with contrast before the procedure. C0, common carotid artery; C1, cervical; C2, petrous; C3, lacerum; C4, cavernous; C5, clinoidal; C6, ophthalmic; and C7, communicating segment of internal carotid artery.

The HRVWI was done using a double inversion recovery technique and 3D motion sensitized driven equilibrium rapid gradient echo (3D-MERGE) technique with contrast. The lesion locations were recorded according to the ICA classification proposed by Bouthillier et al. (9), in which the ICA is divided into seven segments, i.e., C1, cervical; C2, petrous; C3, lacerum; C4, cavernous; C5, clinoidal; C6, ophthalmic; and C7, communicating. The axial images would be reviewed, and independent neurosurgeons and radiologists would measure the diameter of the occluded ICA from C1 to C7 (Figure 1).

### Treatment

All the patients would be given regular medical treatment including management of risk factors (elevated systolic BP, elevated low-density lipoprotein cholesterol level, diabetes mellitus, smoking, etc.). Aspirin (100 mg per day) and clopidogrel (75 mg per day) would be prescribed to the patient for more than 7 days. Patients were treated by either carotid endarterectomy (CEA) or hybrid surgery (Supplementary Table 1). The procedure was performed by experienced doctors in the operating room equipped with an angiographic fluoroscopy system (FD20 system, Philips, Germany). Patients treated by CEA would have a plaque resection and arterial catheter embolectomy with a Forgaty catheter under the microscope first. Cerebral angiography would be done after that. While for patients treated by hybrid surgery, endovascular recanalization would be further performed. The target common carotid artery was engaged with 8-F MPA guiding catheter. Guidewires and microcatheters were used to explore the distal true lumen of occluded ICA and try to re-enter it. After the microwire entered the distal true lumen, the microcatheter would be exchanged to a properly sized balloon. Pre-dilation with a balloon catheter would be performed for distal to proximal. Cerebral angiography would be done to confirm the recanalization. If severe residual stenosis or dissection was found, properly sized balloon-mounted stents for distal segment lesion and carotid artery self-expanding stents for proximal segment lesion would be done at that time if necessary. The sequential endovascular treatment was abandoned after 30 min of futile effort, or when the wire tip is confirmed to be extravascular.

### Statistical Analysis

Statistical analysis is performed with SPSS 22.0 software. Categorical variables are described in numbers and percentages. Continuous variables are expressed as mean ± standard deviation (SD). The chi-square test or Fisher's exact test is used to compare groups of categorical data. The relationships between HRVWI appearance and recanalization were assessed using Logistic regression. ROC curve was used to compare the predictive value HRVWI in recanalization surgery. The score-based prediction model was generated from the logistic regression equations by using a regression coefficient-based scoring method (10). A two-sided *p*-value of 0.05 was considered statistically significant.

## Results

A total of 31 patients were enrolled in the present study. The clinical characteristics of patients are summarized in Table 1. The male-to-female ratio is 5.2:1 (26 males: 5 females). The overall success rate of recanalization is 67.7% (21/31). Among the 31 patients, many cases had chronic comorbidities such as hypertension (59.3%), diabetes (34.4%), coronary artery disease (18.8%), and hyperlipidemia (50.0%). Meanwhile, 10 (31.3%) had a history of smoking and 7 (21.9%) have a history of drinking. Two of the 31 cases had a history of ICA stenting, one in the ipsilateral side and one in the contralateral side, and both of them failed in recanalization. No significant differences are found between the success and failure cases with regard to the comorbidities, history of smoking or drinking, and most of the biochemical indexes.

**Table 1:** Clinical characteristics of patients.

	**Success cases** **(*N* = 21)**	**Failure cases** **(*N* = 10)**	**Total** **(*N* = 31)**	***p*-value**
Male, %	20 (95.2)	6 (60.0)	26 (83.9)	0.027^*^
Age, years	61.57 ± 8.95	63.80 ± 10.56	62.81 ± 9.69	0.545
Hypertension, %	14 (63.6)	5 (50.0)	19 (61.3)	0.447
Diabetes, %	6 (27.3)	5 (50.0)	11 (35.4)	0.423
CAD, %	2 (9.1)	4 (40.0)	6 (19.4)	0.067
Hyperlipidemia, %	11 (50.0)	5 (50.0)	16 (51.6)	1.000
TC, mmol/L	3.11 ± 0.74	3.42 ± 0.79	3.21 ± 0.74	0.314
LDL, mmol/L	1.73 ± 0.56	1.84 ± 0.52	1.77 ± 0.53	0.613
HDL, mmol/L	0.91 ± 0.21	0.96 ± 0.25	0.93 ± 0.22	0.599
VLDL, mmol/L	0.50 ± 0.16	0.72 ± 0.32	0.56 ± 0.24	0.072
Triglyceride, mmol/L	1.20 ± 0.44	1.64 ± 1.00	1.34 ± 0.67	0.100
Homocysteine, μmol/L	12.58 ± 3.50	11.81 ± 2.06	12.52 ± 3.21	0.545
CRP, mg/L	3.90 ± 6.37	4.78 ± 6.13	4.04 ± 6.14	0.731
Smoking, %	8 (36.4)	2 (20.0)	10 (32.3)	0.677
Drinking, %	5 (27.3)	1 (10.0)	7 (22.6)	0.634
History of ICA stenting				0.106
Ipsilateral, %	0	1 (10.0)	1 (3.2)	
Contralateral, %	0	1 (10.0)	1 (3.2)	

*Values are mean ± SD or N (%)*.

* *p < 0.05*;

*CAD, coronary artery disease; TC, total cholesterol; LDL, low density lipoprotein; HDL, high density lipoprotein; VLDL, very low density lipoprotein; CRP, C reactive protein*.

The clinical characteristics of the lesions are summarized in Table 2. No significant differences are found between the success and failure cases in terms of occlusion length, the distal end of the occluded segment, and the treatment applied.

**Table 2:** Clinical characteristics of lesions.

	**Success cases** **(*N* = 21)**	**Failure cases** **(*N* = 10)**	**Total** **(*N* = 31)**	***p*-value**
Right side, %	12 (57.1)	8 (80.0)	20 (64.5)	0.214
Occlusion length				0.363
≥50 mm, %	16 (76.2)	9 (90.0)	25 (80.6)	
<50 mm, %	5 (23.8)	1 (10.0)	6 (19.4)	
Distal end of occluded segment				0.145
Proximal to C5, %	16 (76.2)	5 (50.0)	21 (67.7)	
Distal to or at C5, %	5 (23.8)	5 (50.0)	10 (32.3)	
Type of occlusion				0.704
Total occlusion, %	19 (90.5)	10 (100.0)	29 (93.5)	
Near-occlusion, %	2 (9.5)	0	2 (6.5)	
Treatment				0.067
Hybrid, %	12 (57.1)	9 (90.0)	21 (65.6)	
CEA, %	9 (42.9)	1 (10.0)	10 (31.3)	

*Values are N (%). C3, lacerum segment; C4, cavernous segment; C5, clinoidal segment; CEA, carotid endarterectomy; ICA, internal carotid artery*.

In all of the 31 cases, the lesions began at C1. Therefore, the occluded ICA could be separated into two parts, including the proximal occlusive part beginning at C1, and the distal non-occlusive part ending at C7. The mean ipsilateral-to-contralateral diameter ratios (I/C ratios) of each part is calculated as the geometric mean of the I/C ratio of each segment it contains. As Table 3 showed, significant differences between groups are found in the I/C ratio of the occlusive part (*p* < 0.001), but not in that of the non-occlusive part distal to the lesion.

**Table 3:** The I/C ratio and diameter of the occluded internal carotid artery.

	**(Mean) I/C ratio**			**Diameter**		
	**Success** **(*N* = 22)**	**Failure** **(*N* = 10)**	***p*-value**	**Success** **(*N* = 22)**	**Failure** **(*N* = 10)**	***p*-value**
Occlusive part	0.89 ± 0.06	0.77 ± 0.07	<0.001^‡^	/	/	/
Non-occlusive part	0.89 ± 0.08	0.72 ± 0.28	0.095	/	/	/
C1	0.93 ± 0.12	0.79 ± 0.15	0.009^†^	0.44 ± 0.10	0.43 ± 0.09	0.646
C2	0.93 ± 0.13	0.70 ± 0.16	<0.001^‡^	0.42 ± 0.09	0.36 ± 0.10	0.090
C3	0.88 ± 0.09	0.83 ± 0.10	0.177	0.39 ± 0.12	0.42 ± 0.09	0.516
C4	0.89 ± 0.10	0.82 ± 0.22	0.190	0.35 ± 0.10	0.36 ± 0.09	0.864
C5	0.87 ± 0.08	0.75 ± 0.28	0.236	0.28 ± 0.08	0.25 ± 0.07	0.303
C6	0.85 ± 0.12	0.68 ± 0.29	0.098	0.24 ± 0.06	0.20 ± 0.06	0.053
C7	0.93 ± 0.11	0.78 ± 0.34	0.188	0.23 ± 0.07	0.18 ± 0.05	0.032^*^
ACA	1.00 ± 0.63	1.02 ± 0.76	0.917	0.16 ± 0.05	0.17 ± 0.07	0.746
MCA	0.87 ± 0.34	0.82 ± 0.28	0.748	0.22 ± 0.04	0.21 ± 0.06	0.477

*Values are mean ± SD*.

* *p < 0.05*;

† *p < 0.01*;

‡ *p < 0.001*;

*I/C ratio, ipsilateral-to-contralateral diameter ratio; C1, cervical segment; C2, petrous segment; C3, lacerum segment; C4, cavernous segment; C5, clinoidal segment; C6, ophthalmic segment; C7, communicating segment; ACA, anterior cerebral artery; MCA, middle cerebral artery*.

We also compared the diameter and the I/C ratio of each segment of ICA between cases that succeeded or failed in recanalization (Table 3). In C1 and C2 of the occluded ICA, significant differences are found in the I/C ratio (*p* = 0.009, *p* < 0.001). Meanwhile, a smaller diameter of C7 of the occluded ICA is significantly related to the failure of recanalization (*p* = 0.032).

**Figure 2:**
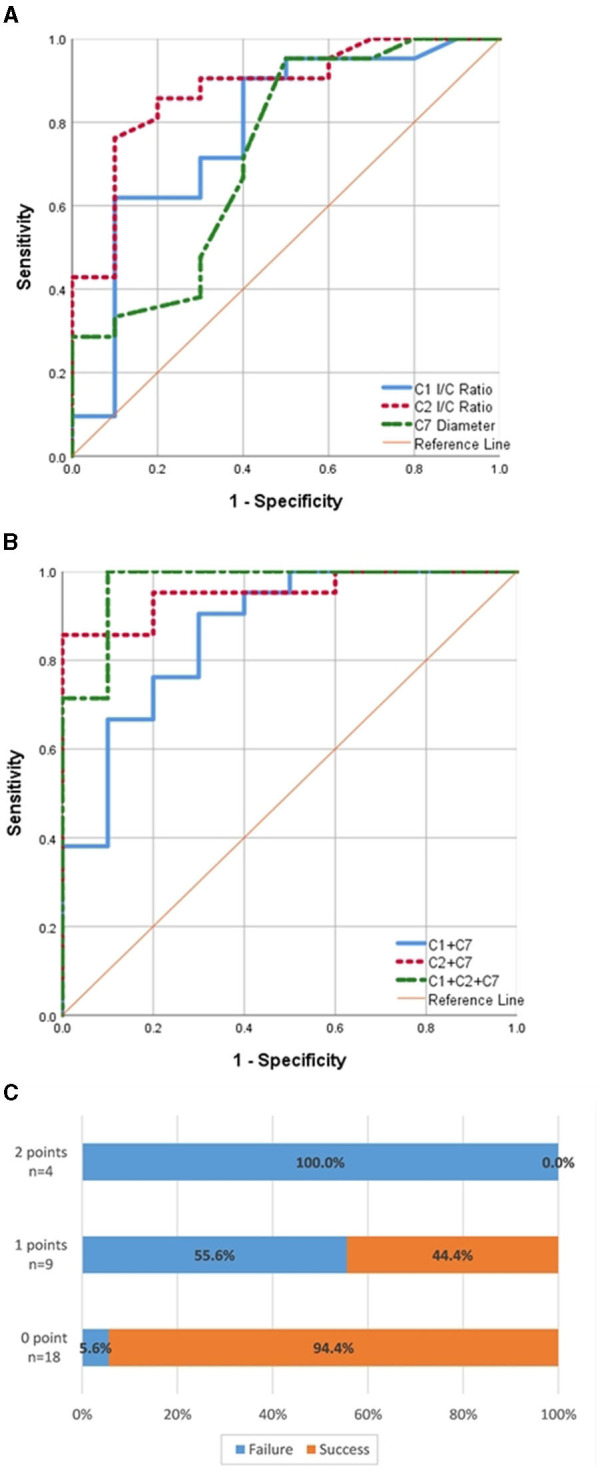
**(A)** Receiver operating characteristic (ROC) curves to describe the performance of the I/C ratio of C1 or C2, the diameter of C7 in predicting the success of recanalization surgery. **(B)** ROC curves to describe the performance of the combination of the I/C ratio of C1 and the diameter of C7 (C1 + C7), the I/C ratio of C2 and the diameter of C7 (C2 + C7), the I/C ratio of C1 and C2 and the diameter of C7 (C1 + C2 + C7) in predicting the success of recanalization surgery. **(C**) Effects of the presence of 0–2 predictive factors associated with the failure or success of recanalization. y-Axis shows each group of patients scoring 0, 1, or 2 points and the actual number of patients is marked. x-Axis shows percentage of patients who failed or succeeded in recanalization in each group.

ROC curves were plotted to evaluate the predictive value of the I/C ratios of C1 or C2, the diameter of C7, and certain combinations of the factors mentioned above (Figure 2). The results showed that the most efficient factor is the I/C ratio of C2 (AUC = 0.876, optimal cut-off = 0.86), while the I/C ratio of C1 and the diameter of C7 also bear moderately good efficiency (AUC = 0.779, optimal cut-off = 0.82; AUC = 0.729, optimal cut-off = 0.175). Among various combinations, the combination of data of C1, C2, and C7 show the highest efficiency (AUC = 0.971), closely followed by the combination of C2 and C7 (AUC = 0.952).

Considering strong collinearity between the data of C1 and C2 and the limited sample capacity, we omitted C1 and chose the data of C2 and C7 to construct a two-factor risk stratification model. In the multivariate analysis, the I/C ratio of C2 <0.86 (OR = 19.814; 95% CI: 1.657–236.887) and diameter of C7 <1.75mm (OR = 42.720; 95% CI: 2.276–801.860) are both independently associated with recanalization failure. We assigned each variable 1 point as a risk score according to their β coefficient in logistic regression analysis (Table 4). The patients scored as 0, 1, or 2 points separately bear a failure risk of 5.6% (1/18), 55.6% (5/9), or 100% (4/4) (Figure 2C).

**Table 4:** ICAO score and success rate of recanalization surgery.

	**OR**	**95% CI**	**β coefficient**	**Point assigned**	**p-value**
C2 I/C ratio < 0.86	19.8	1.7–236.9	2.986	1	0.018^*^
C7 diameter < 0.175	42.7	2.3–801.9	3.755	1	0.012^*^

* *p < 0.05*;

*OR, odds ratio; CI, confidential interval; C2, petrous segment; C7, communicating segment; I/C Ratio, ipsilateral-to-contralateral diameter ratio; ICAO, internal carotid artery occlusion*.

Within 2 weeks postoperatively, three male patients experienced TIA, among whom two succeeded in recanalization while one failed. No patients experienced death, stroke, hemorrhagic transformation, hyperperfusion syndrome, or other severe complications.

## Discussion

Optimal managements for internal carotid artery occlusion continue to be debated. Recent clinical trials showed that endovascular treatment could make a noticeable difference in the natural outcomes of patients with ICAO (11), including carotid endarterectomy (CEA) and carotid artery stenting (12–15). However, endovascular recanalization of ICAO is technically challenging. The visual clues for wiring across the occlusion, such as bridging collateral or distal artery reconstitution, are often lacking, and the potential complications after failing to wire through the right course, including hemorrhage, pseudoaneurysm, and carotid-cavernous fistula, could lead to severe consequences. Therefore, a predictive model is important to identify patients that carry higher success rates and are more suitable for the surgery.

In the present study, we found that the mean ipsilateral-to-contralateral diameter ratio (I/C ratio) of the proximal occlusive part of ICA, which might reflect its degree of atrophy, is significantly associated to the result of recanalization surgery. However, this is not the case when it comes to the mean I/C ratio of the distal non-occlusive part of ICA. The probable reason is that the perfusion from collateral circulation, which typically involves Willis circle or ophthalmic artery, etc. (16), protects the distal ICA segments against further atrophy.

We further analyzed the data of each single segment and found that the I/C ratios of C1 and C2 are positively related to recanalization procedure. According to previous studies, longer occlusion duration has a negative impact on the success rate of recanalization (5, 17). However, since clinical symptoms might not appear synchronically with the onset of ICAO, and there is hardly any image of ICAO obtained as evidence before clinical diagnosis, it is very difficult to determine the exact occlusion duration. The majority of ICAO cases are caused by atherosclerosis, especially in old patients (17, 18). As the occlusion duration gets longer, the thrombus gradually becomes fibrotic or calcified, and the occluded segments of ICA undergoes atrophy. In this case, the difficulty of wiring through the lesion will increase, so will the risk for development of potential complications, e.g., arterial dissection (19, 20). The atherosclerotic lesion typically develops from C1 (5), which marks the bifurcation of the common carotid artery. The plaque forms under low shear stress and slow blood flow here, then gradually extends toward the distal of ICA (21, 22). Given the facts above, we assumed that C1 and C2 segments are usually affected in the early stage of ICAO and the ipsilateral-to-contralateral diameter ratios of C1 and C2 reflect the atrophy degree of the affected segments and, therefore, indirectly indicate the occlusion duration of ICA and influence the success rate of recanalization.

However, the difference could not be detected between success and failure cases in the case of diameters of C1 and C2 (*p* = 0.687; *p* = 0.098). Liu et al. (23) also reported that the proximal occlusion diameter did not show any impact on the success rate of recanalization by CEA. The probable reason for it might be that the individual differences in vascular diameters masked the degree of atrophy.

On the other hand, our study demonstrated that the diameter of C7 is also positively related to the success rate of recanalization surgery. Similar results are reported by Liu et al. (23), that no success of recanalization by CEA was achieved in ICAO cases with a distal occlusion diameter of ICA < 3mm. We assumed that the diameter of the distal end of ICA might reflect perfusion pressure and the quality of collateral circulation. In most chronic ICAO patients, cerebral vascular collateral circulation can be established due to chronic hypoperfusion caused by arterial flow restrictions (16). With contrast injection from collateral pathways, a better vision of the distal ICA could be obtained, which provides clues of “road-map” in guidewire directing.

Based on the data we obtained, we constructed a two-factor model to predict the success of recanalization surgery, involving the I/C ratio of C2 and the diameter of C7, which separately reflect ICAO features in different aspects discussed above. In the 31 cases we have studied, the present model achieved a great efficiency in risk stratifying. Patients who got 2 points in this scoring system achieved no success in recanalization surgery (0/4), while most of the patients who got 0 point achieved successful recanalization (17/18).

Limitations of our research are evident. First, our sample capacity was relatively small, which merely reached the least requirement for logistic regression analysis. The formula we deduced to predict the success rate of recanalization should be further validated or revised in the future study of a larger group of ICAO cases. Second, this is a retrospective study and we only collected the data of the ICAO patients with available HRVMI results, which might cause selection bias.

In conclusion, to the best of our knowledge, this was the first study to clarify the association between the success rate of recanalization surgery and the vascular diameter data of ICA in ICAO patients. The predictive model we constructed can provide useful information in discriminating the population suitable for recanalization surgery.

## Data Availability Statement

The raw data supporting the conclusions of this article will be made available by the authors, without undue reservation.

## Ethics Statement

The studies involving human participants were reviewed and approved by the Ethics Committee of Peking University International Hospital. The patients/participants provided their written informed consent to participate in this study.

## Author Contributions

CY: original idea and data collection. RG and JW: data analysis and article writing. WJ and YZ: data collection. RW: article revision. All authors contributed to the article and approved the submitted version.

## Conflict of Interest

The authors declare that the research was conducted in the absence of any commercial or financial relationships that could be construed as a potential conflict of interest. The reviewer NM declared a shared affiliation with one of the authors RW to the handling editor at time of review.

## Publisher's Note

All claims expressed in this article are solely those of the authors and do not necessarily represent those of their affiliated organizations, or those of the publisher, the editors and the reviewers. Any product that may be evaluated in this article, or claim that may be made by its manufacturer, is not guaranteed or endorsed by the publisher.

## Abbreviations

CEA: carotid endarterectomy
HRVWI: high-resolution vessel wall magnetic resonance imaging
ICA: internal carotid artery
ICAO: internal carotid artery occlusion
I/C ratios: ipsilateral-to-contralateral diameter ratios.
