# Supplementary material for: Vascular Diameters as Predictive Factors of Recanalization Surgery Outcomes in Internal Carotid Artery Occlusion
Source: Front Neurol. 2021 Sep 6;12:632063. doi: 10.3389/fneur.2021.632063 (PMC8451719; doi:10.3389/fneur.2021.632063)
Supplement: Supplementary file 1 [file Table_1.DOCX]

**Supplemental Table 1. ICAO Patients List**

ICAO Patients List. Basic data of patients enrolled.

| Name Initials | Sex | Age | Procedure | Location of Lesion |
| --- | --- | --- | --- | --- |
| WA | Male | 74 | CEA | C1-C2 |
| XF | Male | 77 | CEA | C1-C2 |
| WHD | Male | 74 | CEA | C1-C2 |
| DWG | Male | 52 | CEA | C1-C2 |
| ZYC | Male | 69 | CEA | C1-C2 |
| FCM | Male | 53 | Hybrid | C1-C3 |
| WP1 | Male | 59 | Hybrid | C1-C3 |
| MYC | Male | 69 | Hybrid | C1-C3 |
| LHP | Male | 79 | Hybrid | C1-C3 |
| RPJ | Male | 43 | Hybrid | C1-C3 |
| ZXY | Male | 77 | Hybrid | C1-C4 |
| XFS | Male | 59 | Hybrid | C1-C4 |
| CJP | Male | 62 | Hybrid | C1-C4 |
| ZSJ | Male | 51 | Hybrid | C1-C4 |
| WDH | Male | 62 | Hybrid | C1-C4 |
| WYS | Male | 53 | Hybrid | C1-C4 |
| XYL | Male | 70 | Hybrid | C1-C4 |
| LZL | Male | 69 | Hybrid | C1-C4 |
| LSG | Male | 65 | CEA | C1-C4 |
| CHF | Male | 61 | CEA | C1-C4 |
| BS | Male | 53 | CEA | C1-C4 |
| YSQ | Female | 69 | Hybrid | C1-C5 |
| WHQ | Male | 63 | Hybrid | C1-C5 |
| ZDC | Male | 58 | Hybrid | C1-C5 |
| QGL | Female | 64 | Hybrid | C1-C5 |
| WP2 | Female | 43 | Hybrid | C1-C5 |
| CXY | Female | 71 | Hybrid | C1-C5 |
| JHS | Male | 63 | Hybrid | C1-C6 |
| WSX | Male | 60 | Hybrid | C1-C6 |
| CXL | Female | 77 | CEA | Near-occlusion (C1-C7) |
| LPH | Male | 53 | CEA | Near-occlusion (C1-C7) |
| CEA = Carotid Endarterectomy; C1=cervical segment; C2=petrous segment; C3=lacerum segment; C4=cavernous segment; C5=clinoidal segment; C6=ophthalmic segment | | | | |
